# *Plasmodium berghei* Brca2 is required for normal development and differentiation in mice and mosquitoes

**DOI:** 10.1186/s13071-022-05357-w

**Published:** 2022-07-08

**Authors:** Yasunaga Yoshikawa, Shunta Kimura, Akira Soga, Makoto Sugiyama, Aki Ueno, Hiroki Kondo, Zida Zhu, Kazuhiko Ochiai, Kazuhiko Nakayama, Jun Hakozaki, Kodai Kusakisako, Asako Haraguchi, Taisuke Kitano, Koichi Orino, Shinya Fukumoto, Hiromi Ikadai

**Affiliations:** 1 Laboratory of Veterinary Biochemistry, School of Veterinary Medicine, Kitasato University, Towada, Aomori 034-8628 Japan; 2 National Research Center for Protozoan Diseases, Obihiro University of Agriculture and Veterinary Medicine, Inada, Obihiro 080-8555 Japan; 3 Laboratory of Veterinary Anatomy, School of Veterinary Medicine, Kitasato University, Towada, Aomori 034-8628 Japan; 4 Laboratory of Veterinary Hygiene, School of Veterinary Medicine, Nippon Veterinary and Life Science University, Musashino, Tokyo 180-8602 Japan; 5 Laboratory of Veterinary Parasitology, School of Veterinary Medicine, Kitasato University, Towada, Aomori 034-8628 Japan

**Keywords:** Breast cancer susceptibility protein 2, Homologous recombination, Meiosis, *Plasmodium*, DNA repair protein Rad51 homolog 1

## Abstract

**Background:**

Malaria is a major global parasitic disease caused by species of the genus *Plasmodium*. Zygotes of *Plasmodium* spp. undergo meiosis and develop into tetraploid ookinetes, which differentiate into oocysts that undergo sporogony. Homologous recombination (HR) occurs during meiosis and introduces genetic variation. However, the mechanisms of HR in *Plasmodium* are unclear. In humans, the recombinases DNA repair protein Rad51 homolog 1 (Rad51) and DNA meiotic recombinase 1 (Dmc1) are required for HR and are regulated by breast cancer susceptibility protein 2 (BRCA2). Most eukaryotes harbor BRCA2 homologs. Nevertheless, these have not been reported for *Plasmodium*.

**Methods:**

A Brca2 candidate was salvaged from a database to identify Brca2 homologs in *Plasmodium*. To confirm that the candidate protein was Brca2, interaction activity between *Plasmodium berghei* (Pb) Brca2 (PbBrca2) and Rad51 (PbRad51) was investigated using a mammalian two-hybrid assay. To elucidate the functions of PbBrca2, *PbBrca2* was knocked out and parasite proliferation and differentiation were assessed in mice and mosquitoes. Transmission electron microscopy was used to identify sporogony.

**Results:**

The candidate protein was conserved among *Plasmodium* species, and it was indicated that it harbors critical BRCA2 domains including BRC repeats, tower, and oligonucleotide/oligosaccharide-binding-fold domains. The *P. berghei* BRC repeats interacted with PbRad51. Hence, the candidate was considered a Brca2 homolog. *PbBrca2* knockout parasites were associated with reduced parasitemia with increased ring stage and decreased trophozoite stage counts, gametocytemia, female gametocyte ratio, oocyst number, and ookinete development in both mice and mosquitoes. Nevertheless, the morphology of the blood stages in mice and the ookinete stage was comparable to those of the wild type parasites. Transmission electron microscopy results showed that sporogony never progressed in *Brca2*-knockout parasites.

**Conclusions:**

Brca2 is implicated in nearly all *Plasmodium* life cycle stages, and especially in sporogony. PbBrca2 contributes to HR during meiosis.

**Graphical Abstract:**

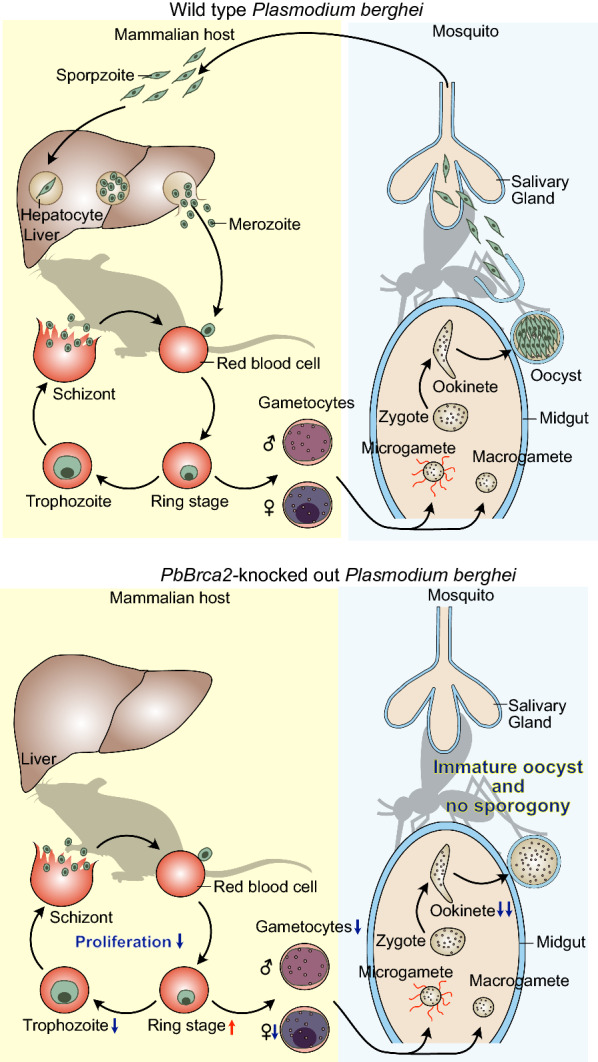

**Supplementary Information:**

The online version contains supplementary material available at 10.1186/s13071-022-05357-w.

## Introduction

Malaria is an infection caused by protozoan parasites of the genus *Plasmodium*. In 2020, there were an estimated 229 million cases of malaria and 409,000 deaths caused by the disease worldwide [World Health Organization (WHO): https://www.who.int/teams/global-malaria-programme/reports/world-malaria-report-2020]. Malaria is difficult to control, and no efficacious vaccine has been developed against it. Moreover, there is continuous antigenic variation, genetic diversity, and antimalarial drug resistance in *Plasmodium* [1, 2]. In humans, *Plasmodium* is transmitted from host to host via *Anopheles* mosquitoes [3]. When a mosquito takes a blood meal from a host, it injects *Plasmodium* sporozoites into the bloodstream. The sporozoites ultimately migrate to the host’s liver where they develop into merozoites, which are then released into the circulatory system and start an asexual reproduction cycle. Only a small proportion of the parasites exist as gametocytes in the blood. When a female *Anopheles* mosquito takes a blood meal from a host, gametocytes are ingested, pass into the midgut and differentiate into male and female gametes. Fertilization, zygote formation, and meiosis then occur. Next, the zygotes differentiate into motile ookinetes. The latter migrate, traverse the midgut epithelium, settle under the midgut basal lamina, and differentiate into oocysts at approximately 24 h after blood ingestion. The asexual reproduction cycle then resumes once again. Several thousand sporozoites form within each oocyst and are released approximately 2 weeks after blood ingestion. The sporozoites then migrate and invade the mosquito salivary glands, which enables the insect to transmit the pathogen to another host.

Efficacious drugs targeting all *Plasmodium* life cycle stages are required to control malaria. The zygote to ookinete stages are feasible drug targets as the numbers at these stages are far lower than at the other stages. However, the currently available anti-*Plasmodium* drugs only target blood-stage parasites present in the mammalian host. Sexual reproduction and meiosis occur from the zygote to ookinete stages. Homologous recombination (HR) occurs during meiosis in *Plasmodium* spp., which plausibly involves the DNA recombinases DNA repair protein Rad51 homolog 1 (Rad51) and DNA meiotic recombinase 1 (Dmc1) [3–6]. It was reported that *Dmc1* knockout (KO) in *Plasmodium berghei* resulted in a reduced number and altered development of oocysts and sporozoites [6]. Rad51 and Dmc1 also regulate HR during meiosis in mammals [7]. In mammals they interact with BRCA2 and are regulated by it. This mechanism also contributes to HR during meiosis [7, 8]. Mammalian BRCA2 harbors several functional domains. In humans, Rad51 and Dmc1 interact with BRCA2 through BRC repeats. Rad51 and Dmc1 also interact with BRCA2 through the C-terminal Rad51- and DMC1-binding domains, respectively [9–11]. HR also participates in DNA double-strand break (DSB) repair. Most living organisms repair DSBs via HR and/or non-homologous end joining. The HR-related proteins are conserved in *Plasmodium* whereas the non-homologous end joining proteins are not [3]. Thus, most DSBs are repaired by *Plasmodium* HR proteins [12].

BRCA2 orthologs have been detected in eukaryotes such as mammals, *Xenopus*, fish, *Caenorhabditis elegans*, *Ustilago maydis*, *Trypanosoma*, and *Leishmania*. They are absent in yeasts, archaea, and bacteria [13–17]. In yeasts, Rad52 is the Rad51-regulating protein. Human BRCA2 cannot interact with yeast Rad51 [18]. In lower eukaryotes such as *C. elegans*, *U. maydis*, *Trypanosoma*, and *Leishmania*, Brca2 orthologs possess only the vital BRC repeats and the tower and oligonucleotide/oligosaccharide-binding (OB)-fold domains. In *Plasmodium*, HR proteins, including Rad51 and Dmc1, are conserved and their functions resemble those of human Rad51 and Dmc1 [6]. However, the Rad51 and Dmc1 regulatory protein, such as BRCA2, has not been identified. Thus, the aims of the present study were to salvage BRC repeats and DNA binding domain-containing proteins from the *Plasmodium* database and clarify the functions of *Plasmodium* Brca2 by generating KO *P. berghei*.

## Methods

### Bioinformatics

The *P. berghei* (Pb) *Brca2* (*PbBrca2*) (PbANKA_1343400) genomic sequences used in the present study were retrieved from PlasmoDB (http://www.plasmodb.org). The online hidden Markov model structure prediction tool HHpred (https://toolkit.tuebingen.mpg.de/tools/hhpred) was used to predict the features of the PbBrca2 domain [19, 20]. The online signal peptide prediction tool PSORT II Prediction (https://psort.hgc.jp/form2.html) was used to predict the subcellular localization of PbBrca2 [21, 22].

### Crystal structure modeling

The crystal structures of human Rad51 and BRC repeat 4 were retrieved from the Research Collaboratory for Structural Bioinformatics Protein Data Bank (PDB) at http://www.rcsb.org/ [PDB identifier (ID) 1N0W] and analyzed with Chimera software (http://www.cgl.ucsf.edu/chimera/) [8]. The OB-fold and tower domains of PbBrca2 were predicted with AlphaFold2 [23], and their structures were compared via Chimera against those of human BRCA2 helical, OB, and tower domains (PDB ID 1MIU) or that of the OB domain of human replication protein A1 (PDB ID 4O0A).

### Mice, parasites, and mosquitoes

ICR and BALB/c mice aged 6–8 weeks were obtained from Charles River Laboratories Japan (Yokohama, Kanagawa, Japan). The *P. berghei* ANKA strain constitutively expressing green fluorescent protein was obtained from Dr. M. Yuda (Mie University, Tsu, Mie Prefecture, Japan) [24]. *Anopheles stephensi* (STE2 strain) mosquitoes were kept in an insectary at 27 °C, 80% relative humidity, and a 14-h/10-h light/dark cycle and fed 10% (w/v) sucrose solution. All experimental procedures were executed following a protocol approved by the Kitasato University Animal Care and Use Committee (approval nos. 16-020 and 19-087).

### Mammalian two-hybrid assay

The coding regions of the PbBrca2 BRC repeats domain were cloned into pM plasmids (Clontech Laboratories, Mountain View, CA). *Plasmodium berghei* Rad51 and Dmc1, *Saccharomyces cerevisiae* Rad51, and human Rad51 were cloned into pVP16 plasmids (Clontech Laboratories). The methods used for the mammalian two-hybrid assays have been previously described [25]. FuGENE HD transfection reagent (Promega, Madison, WI) was used to co-transfect ~ 2 × 10^5^ cells 293 T cells with pM and pVP16 vectors, the pGluc luciferase reporter, and pRL-tk normalization plasmids (Promega). The luciferase activity of the cell extracts was measured with a Dual-Luciferase Reporter Assay System (Promega).

### Generation of *PbBrca2*-KO parasites

*PbBrca2* was knocked out by double-crossover HR technology using a pBluescript II-based plasmid containing a puromycin resistance gene inserted upstream (5′) of the *PbEf1α* gene and downstream (3′) of the *PbDhfr-ts* gene, as previously described [26, 27]. A donor plasmid with 1000-bp homology arms was cloned upstream with the primers 5′-*PbBrca2* forward (F) (5′-CGGGGTACCTTTTATTGTATCCCTATAA-3′), and 5′-*PbBrca2* reverse (R) (5′-GGCGGGCCCCTTTATTTAATTATTAAGATTTTTTTGTTAC-3′); and cloned downstream with the primers 3′ *PbBrca2* F (5′-CCGTCTAGATATCGTTTTAAGGTTGTTTC-3′), and 3′-*PbBrca2* R (5′-CCGGCGGCCGCTATGTTGTATTGTTTGTTTT-3′). To clone the homology arms, the plasmids were treated with the restriction enzymes KpnI and ApaI for upstream of the *PbBrca2* gene and XbaI and NotI for downstream of the *PbBrca2* gene (all restriction enzymes were purchased from New England Biolabs, Ipswich, MA). Ten micrograms of donor vector was linearized using ScaI (New England Biolabs) and electroporated with a Nucleofector 2b device (Lonza Group, Basel, Switzerland) into cultured *P. berghei* schizonts. Transfected parasites were selected by puromycin and cloned by limiting dilution as previously described [26, 27].

### *PbBrca2*-KO parasite genotyping and *PbBrca2* amplification in complementary DNA

Blood infected with wild type (WT) or *PbBrca2*-KO parasites was collected and the DNA or RNA extracted with a Gentra Puregene Blood Kit (Qiagen, Düsseldorf, Germany) or a NucleoSpin RNA Blood Kit (TaKaRa Bio, Kusatsu, Shiga, Japan) according to the manufacturers’ instructions. To genotype the *PbBrca2*-KO, the *PbBrca2* locus was amplified by polymerase chain reaction (PCR) using 5′-*PbBrca2* F and 3′-*PbBrca2* R from genomic DNA. The extracted RNA was reverse-transcribed with SuperScript IV VILO Master Mix (Thermo Fisher Scientific, Waltham, MA) according to the manufacturer’s instructions. *PbBrca2* expression was detected by PCR using *PbBrca2* exon 3 F, 5′-GGGAACCACATTTTTAAATGA-3′, and *PbBrca2* exon 4 R, 5′-CCTTTGGGTATGTTCTTAGGG-3′.

### Southern blot analysis

Two micrograms of the extracted DNA was digested with the restriction endonuclease *Eco*RV (Roche Diagnostics, Basel, Switzerland), separated on a 0.8% agarose gel, transferred to a Hybond N^+^ membrane (GE Healthcare, Little Chalfont, UK), and hybridized with probes labeled using a PCR DIG Probe Synthesis Kit (Roche Diagnostics) with the primers *PbPuro* SH F2 (5′-ACCTCGAGAGATCCCGTTTT-3′) and *PbPuro* SH R2 (5′-TTTATGAATCATTGAAGAGACAACA-3′). The signal was detected with alkaline phosphatase-conjugated anti-digoxigenin antibody (Roche Diagnostics) and CDP-Star Detection Reagent (GE Healthcare).

### Phenotypic analysis of *PbBrca2*-KO parasites

The development of WT and *PbBrca2*-KO parasites in the BALB/c mice was assessed. Five mice per group were each injected with 1 × 10^6^ WT or *PbBrca2*-KO parasites. Parasitemia was monitored daily using Giemsa-stained blood smears. The morphology of blood-stage parasites was observed using a phase-contrast microscope (ECLIPSE E200; Nikon, Tokyo). A survival assay was performed using nine and 10 mice infected with WT and *PbBrca2*-KO1 parasites, respectively, for 11 days after infection. The humane endpoints were when mice showed rapid weight loss (> 20% of body weight) or poor physical appearance (reduced mobility, rough coat, and depression). When parasitemia reached approximately 10% in four mice per group, the gametocytes per infected red blood cell were enumerated and the gametocyte sex ratios were determined by Giemsa-stained tail blood smears. Male gametocyte exflagellation was quantified as previously described [28]. Briefly, 1 µL gametocyte-infected blood was drawn from the tail vein and immediately mixed with 1 µL heparin and 38 µL complete ookinete culture medium (OKM; RPMI 1640 medium; Gibco, Grand Island, NY) and 20% (v/v) heat-inactivated fetal bovine serum (Sigma-Aldrich, St. Louis, MO). The mixture was placed under a coverslip at room temperature. After 5 min, the exflagellation centers were counted under a phase-contrast microscope (ECLIPSE E200; Nikon) for the next 10 min. Parasite differentiation into gametocytes and exflagellation were then assessed. *Anopheles stephensi* mosquitoes were fed on the infected mice, and the oocysts were microscopically examined between days 13–15. At least 30 mosquitoes were dissected. Enhanced green fluorescent protein-expressing oocysts were detected by fluorescence stereoscopic microscopy (Leica M205 FA; Leica Microsystems, Wetzlar, Germany). The oocysts in each positive midgut were counted to determine infection intensity and the prevalence of infection [29]. The sporozoites in *A. stephensi* mosquitoes treated as described above were examined microscopically at day 28. Enhanced green fluorescent protein-expressing sporozoites in the salivary glands were detected using fluorescence microscopy (Leica M205 FA; Leica Microsystems).

### Ookinete culture and purification

Blood presenting with 10 × 10^4^ red blood cells in which gametocytes were undergoing exflagellation was obtained from mice by heart puncture. Ten volumes of OKM was added to the blood and the suspension was cultured at 19 °C for 24 h. Ookinetes were enumerated and their structure was examined using culture suspension smears. They were purified in a MidiMACS Separator System (Miltenyi Biotec, Bergisch-Gladbach, Germany) as previously described [28]. A total of 3 mL culture was passed through the system thrice before removing the column from the magnet. The ookinetes were recovered by passing 5 mL OKM through the column. The purified ookinetes were centrifuged at 1000 *g* and 4 °C for 10 min and washed thrice in phosphate-buffered saline. Ookinete morphology was observed by differential interference contrast microscopy (Olympus IX83; Olympus, Tokyo, Japan).

### Transmission electron microscopy

Mosquito midguts were collected at 7, 14, and 21 days post-infection to evaluate oocyst differentiation, sporozoite development, and sporozoite maturation, respectively. The midguts were fixed in 2.5% (v/v) glutaraldehyde and 2% (v/v) paraformaldehyde in 0.03 M HEPES buffer (pH 7.4) at 4 °C for 2 h. The cells were then treated with 1% (w/v) osmium tetrachloride/1.5% (w/v) potassium ferrocyanide at 21–24 °C for 2 h to enhance cytoplasmic contrast [30]. The midguts were then dehydrated, embedded in epoxy resin, sectioned to 70-nm thickness with an Ultracut N (Reichert–Nissei, Vienna), stained with 1% (w/v) uranyl acetate for 18 min and 2% (w/v) lead citrate for 1 min, and examined under a transmission electron microscope (H-7650; Hitachi, Tokyo) at 80 kV [29].

### Statistical analyses

The *F*-test followed by Student’s *t*-test with or without Holm’s correction was used to compare treatments in terms of interaction activity (mammalian two-hybrid assay), parasitemia, infection intensity (number of oocysts/midgut), gametocytemia, gametocyte and exflagellation ratios, and ookinete numbers. A log-rank (Mantel–Cox) test was used to compare the survival rate in the survival assay. The total number of each blood-stage counts and the total number of female and male gametocyte counts were analyzed by a χ^2^-test. The number of oocysts was assessed by a Mann–Whitney *U*-test.

## Results

### Bioinformatic analyses of *Plasmodium* Brca2

**Fig. 1:**
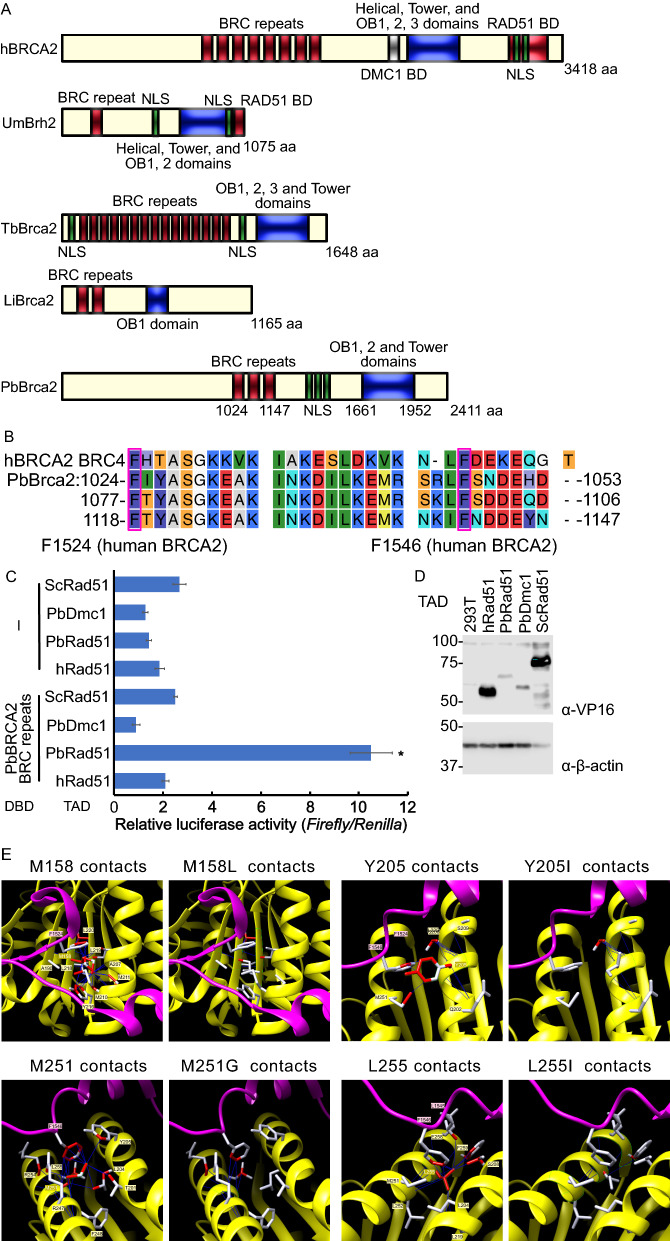
**A–E** Breast cancer susceptibility protein 2 (Brca2) is conserved in *Plasmodium berghei* (Pb). **A** Schematic representation of PbBrca2 protein domains relative to human, *Trypanosoma brucei*, and *Leishmania infantum* BRCA2 and *Ustilago maydis* Brh2. Predicted BRC repeats, tower and oligonucleotide/oligosaccharide-binding (*OB*)-fold domains, and nuclear localization signals are shown. For human BRCA2, PALB2, DMC1, and DNA repair protein Rad51 homolog 1 (Rad51)-binding domains (*BD*) are shown. **B** Alignment of human BRCA2 BRC repeat 4 and three PbBrca2 BRC repeats. Magenta boxes indicate that the phenylalanine residues F1524 and F1546 are required for interaction with Rad51. **C** Interaction activity of PbBrca2 BRC repeats with *P. berghei*, human, and *Saccharomyces cerevisiae* Rad51 and *P. berghei* Dmc1 was tested by mammalian two-hybrid assay; 293T cells were transfected with DNA-binding domain fused with PbBrca2 BRC repeats, transactivation domain (TAD) fused with Rad51 or Dmc1, and luciferase reporter and normalization plasmids. Relative luciferase activity normalized with *Renilla* luciferase activity. Comparison of mean values was performed using the *F*-test followed by Student’s *t*-test; * *P* < 0.05 (vs. paired TAD-fused protein-expressing samples). **D** Expression levels of TAD-fused proteins in mammalian two-hybrid assay were confirmed. All TAD-fused proteins are expressed. **E** Structural representation of the amino acid substitutions in human Rad51. The substituted amino acids are important for interaction with human BRC repeat 4 and differ from PbRad51. The three-dimensional structure based on human Rad51 and BRC repeat 4 crystal structure was predicted using Chimera software. Number of contacts between BRC repeat 4 and Rad51 decrease in the case of all four substitutions

Analysis of the *Plasmodium* genome by the online hidden Markov model structure prediction tool (https://toolkit.tuebingen.mpg.de/tools/hhpred) suggested that the conserved gene product PBANKA_1343400 is a BRCA2 ortholog in *P. berghei* (Fig. 1a). According to PlasmoDB, PBANKA_1343400 is a conserved *Plasmodium* protein with unknown function (https://plasmodb.org/plasmo/app/search?q=PBANKA_1343400). The regions of this gene product that are conserved among *Plasmodium* spp. are presented in Additional file 1: Fig. S1. It was predicted that Brca2 in *P. berghei* has three BRC repeats and OB-fold and tower domains such as *Trypanosoma brucei* and *Leishmania infantum* Brca2 and *Ustilago maydis* Brh2, which are the simplest BRCA2 orthologs harboring essential BRCA2 domains, i.e. one BRC repeat, OB and tower domains, and nuclear localization signals (Fig. 1a). The three-dimensional structure of the predicted PbBrca2 OB-fold and tower domains was generated by AlphaFold2 (https://www.alphafold.ebi.ac.uk) and compared against human BRCA2 and the typical OB domain of the structure of human replication protein A1 (Additional file 2: Fig. S2). AlphaFold2 predicted one tower and two OB domains. We also estimated the subcellular localizing signal using the online subcellular localization prediction tool PSORT II Prediction (https://psort.hgc.jp/form2.html). It was predicted that PbBrca2 has three nuclear localization signals and that it is localized in the nucleus with a probability of 69.6%.

### PbBrca2 BRC repeats interacted only with PbRad51

BRC repeats are essential for human BRCA2 to be able to interact with Rad51. Three predicted BRC repeats of PbBrca2 aligned with human BRCA2 BRC repeat 4 (Fig. 1b). All three BRC repeats of PbBrca2 were highly conserved with human BRC repeat 4. The phenylalanine residues F1524 and F1546 in human BRCA2, which were essential for the Rad51 interaction, were also conserved in PbBrca2 [31]. We used the mammalian two-hybrid assay to confirm that the predicted PbBrca2 BRC repeats interact with PbRad51. Human BRCA2 interacts with Rad51 and DMC1 via BRC repeats and the other binding domains. It does not interact with *S. cerevisiae* Rad51 [9–11, 18]. We tested the interaction activity of the PbBrca2 BRC repeats against *P. berghei*, human, and *S. cerevisiae* Rad51 and the meiosis-specific Rad51 paralog *P. berghei* Dmc1 (Fig. 1c, d). Relative luciferase activity increased only in the *P. berghei* BRC repeats and PbRad51 in the transfected samples (Fig. 1c). The other combination had the same luciferase activity as the negative control which was only transfected with Rad51- or Dmc1-expressing plasmids. We also confirmed Rad51 and Dmc1 expression in each species via the mammalian two-hybrid assay (Fig. 1d). Thus, the PbBrca2 BRC repeats interacted only with PbRad51, not with human Rad51. We aligned the amino acid sequences of *P. berghei* and human Rad51 to assess why interaction activity was observed only in PbBraca2 and PbRad51 (Additional file 3: Fig. S3). The Rad51 homology between *P. berghei* and human Rad51 was > 80%. However, the chemical properties of the amino acid residues M158, Y205, M251, and L255 in human Rad51 differed between human and *P. berghei* Rad51. These residues were associated with the important phenylalanines F1524 and F1546 in the BRC repeats. We predicted the structure using Chimera software, human BRC repeat 4, and the Rad51 three-dimensional structure, and estimated the interaction mechanism (PDB ID 1N0W) (Fig. 1e). We conducted an in silico analysis to estimate the effects of the *P. berghei* amino acid residues on the interaction between human Rad51 and BRC repeat 4. Substitution of M158L, Y205I, M251G, and L255I reduced the number of contacts between BRC repeat 4 and Rad51 (Fig. 1e).

### PbBrca2-KO parasite production

**Fig. 2:**
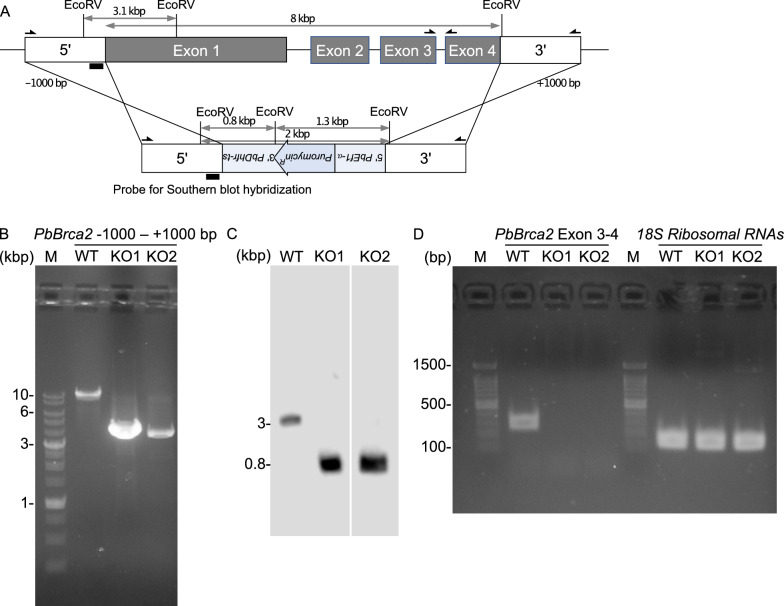
**A–D** Generation of *PbBrca2*-knockout (KO) parasites. **A** Schematic representation of targeted *PbBrca2* disruption. Targeting vector (*bottom*) containing puromycin resistance gene (*Puromycin*^*R*^) upstream (5′) of *PbEf1α* and downstream (3′) of *PbDhfr-ts* was integrated into the *PbBrca2* locus (*top*) by double crossover. Arrows indicate genomic DNA (**B**) and reverse transcription (RT)-polymerase chain reaction (PCR) (**C**) primers. Black bar indicates southern blot probe. **B** *PbBrca2* locus amplified in wild type (*WT*) and *PbBrca2*-KO1 (*KO1*) and *PbBrca2*-KO2 (*KO2*) clones. In the WT parasite, PCR products were longer than those detected for KO1 and KO2 parasites. **C** *PbBrca2* KO was detected by southern blot. *Eco*RV-digested genomic DNA from WT and KO parasites demonstrated hybridization with upstream *PbBrca2*. KO1 and KO2 clones detected relatively short bands. **D** To analyze mRNA-level *PbBrca2* expression, RT-PCR was performed using primers amplifying the region between *PbBrca2* exon 3 and exon 4 in blood-stage parasites. *PbBrca2* PCR products were detected for WT but not for KO1 or KO2 parasites. Amplified 18S ribosomal RNA was the positive control.* M* DNA size marker

Next, we attempted to elucidate the function of PbBrca2. To this end, we generated a *Brca2* null *P. berghei* (Fig. 2). To generate the *Brca2* KO parasite, we transfected a codon-optimized puromycin resistance gene with up- and downstream *PbBrca2* homology arms into a *P. berghei* ANKA strain expressing green fluorescent protein (Fig. 2a). We then subjected the full-length genomic DNA region of the puromycin-selected *PbBrca2* null clones to PCR amplification and southern blot hybridization (Fig. 2b, c). Two *PbBrca2*-KO parasites were generated. A PCR analysis of the genomic DNA revealed that the amplicons of the *PbBrca2*-KO clones (about 4 kbp) were shorter than that of the WT parasite (about 10 kbp). Both KO parasites harbored the predicted genomic DNA fragment size. To confirm the lack of mRNA-level *PbBrca2* expression, we performed reverse transcription-PCR using primers amplifying between exons 3 and 4 of *PbBrca2*. As expected, amplification was absent in the *PbBrca2*-KO parasites (Fig. 2d).

### *PbBrca2* is required for asexual blood-stage growth and oocyst formation

**Fig. 3:**
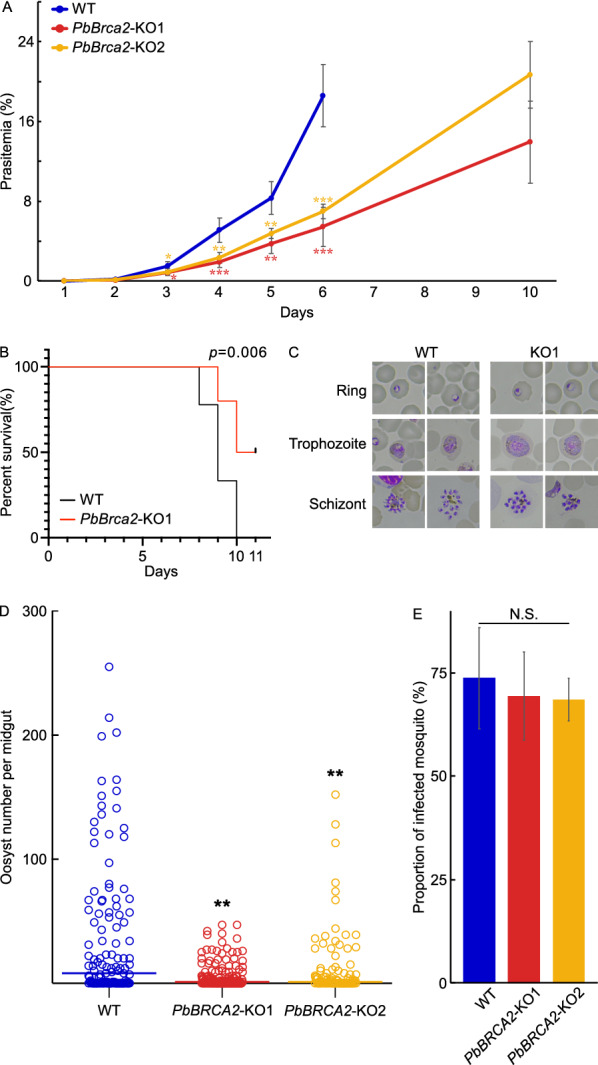
**A–E** PbBrca2 is required for asexual blood-stage growth and oocyst formation. **A** Parasitemia in mice intraperitoneally infected with WT (blue) or *PbBrca2*-KO1 (red) and *PbBrca2*-KO2 (orange) parasites. Each group included five mice, and the mice were infected via intraperitoneal injection with 10^6^ blood-stage parasites. Mice infected with WT and *PbBrca2*-KO parasites were euthanized after days 6 and 10, respectively, in a humane manner. **B** Survival curves of mice infected with WT (*n* = 9) or *PbBrca2*-KO1 (*n* = 10) parasites. Mice demonstrating a highly pathological phenotype were euthanized in a humane manner. A log-rank (Mantel–Cox) test was used to compare survival curves. **C** Morphological analyses of WT and *PbBrca2*-KO1 parasites at the ring, trophozoite, and schizont stages. **D** Oocysts/midgut of mosquitoes infected with WT or *PbBrca2*-KO1 or *PbBrca2*-KO2 parasites. Mosquitoes were fed on three infected mice in each group. Horizontal bars are medians. **e** Proportions of mosquitoes harboring ≥ 1 oocyst. The *F*-test followed by Student’s *t*-test with Holm’s correction was used to compare the mean values of parasitemia and oocyst infection intensity. * *P* < 0.05, ** *P* < 0.01, *** *P* < 0.001,* N.S.* non-significant (vs. WT parasites). Mann–Whitney *U*-test was used to compare the medians of oocyst numbers. For abbreviations, see Figs. 1 and 2

A comprehensive gene expression analysis revealed that *PbBrca2* is upregulated in the schizont, female gametocyte, and ookinete. Moreover, its expression gradually increases from the ring to the schizont stage and gradually decreases from the ookinete to the sporozoite stage (https://www.sanger.ac.uk/science/tools/mca/mca/) [32]. To elucidate the function of *PbBrca2*, we analyzed asexual blood-stage growth (Fig. 3) and found that the *PbBrca2*-KO parasites caused milder parasitemia than the WT (Fig. 3a). The severity of *PbBrca2*-KO parasitemia increased and reached that of WT parasitemia at a later time point. Additionally, we assessed the effect of *PbBrca2*-KO on pathogenesis in mice (Fig. 3b). All the mice infected with WT parasites were euthanized or died within 10 days post-infection, whereas 50% of the mice infected with *PbBrca2*-KO1 parasites were alive at 11 days post-infection, and had a significantly higher survival rate than WT-infected mice (*P* = 0.006). Moreover, mice infected with *PbBrca2*-KO1 parasites maintained their daily level of activity despite high levels of parasitemia. The morphology of the *PbBrca2*-KO parasites at the ring to schizont stages was comparable to that of the WT parasites at the same stages (Fig. 3c); however, the counts at each blood stage differed between the WT and *PbBrca2*-KO parasites (Table 1). Specifically, counts at the ring stage were increased and those at the trophozoite and gametocyte stages were decreased for *PbBrca2*-KO parasites compared to WT parasites.

**Table 1:** Total numbers of parasites at each blood stage

	WT	*PbBrca2*-KO1			*PbBrca2*-KO3		
Ring	243 (60.75 ± 21.23)	357 (71.4 ± 15.82)	**	▲	264 (52.8 ± 24.67)	**	▲
Trophozoite	104 (26 ± 6.98)	34 (6.8 ± 2.68)	**	▽	63 (12.6 ± 10.92)	**	▽
Schizont	40 (10 ± 0)	50 (10 ± 0)	N.S.		50 (10 ± 0)	N.S.	
Gametocyte	65 (16.25 ± 4.27)	44 (8.8 ± 6.46)	*	▽	29 (5.8 ± 4.67)	**	▽

Counts for each blood stage were compared between wild type (*WT*) (*n* = 4) and *Plasmodium berghei* (Pb) breast cancer susceptibility protein 2 (*PbBrca2*)-knockout 1 (*KO1*) (*n* = 5) or -KO2 (*n* = 5) parasite-infected mice. Numbers in parentheses indicate the mean ± SD. Black triangles indicate a significant increase in the count at that stage compared to that of WT, white triangles indicate a significant decrease in the count at that stage compared to that of WT. * *P* < 0.05, ** *P* < 0.01,* N.S.* non-significant (vs. WT parasites, compared using χ^2^-test)

We then analyzed oocyst formation in mosquitoes because they are susceptible to parasite infestation at that stage. Oocyst formation decreased in the *PbBrca2*-KO parasites (Fig. 3d). We evaluated the proportions of mosquitoes infected with more than one oocyst to determine whether the *PbBrca2*-KO parasites had low infectivity in the mosquitoes. There was no difference in the severity of midgut infestation caused by the WT and *PbBrca2*-KO parasites (Fig. 3e).

### Female gametocyte and ookinete formation are inhibited in *PbBrca2*-KO parasites

**Fig. 4:**
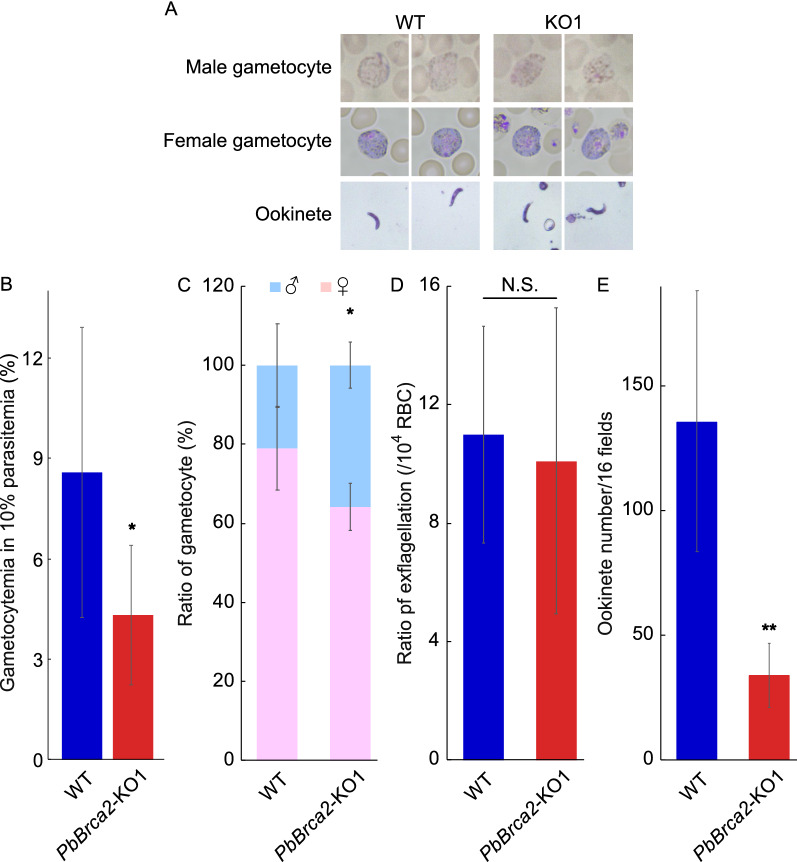
**A–E** PbBrca2 is vital for female gametocyte development and ookinete formation. **A** Morphological analyses of male and female gametocytes and ookinetes of WT and *PbBrca2*-KO1 parasites. **B** Gametocytes were enumerated in four mice infected with WT or *PbBrca2*-KO1 parasites at approximately 10% parasitemia. **C** Proportions of female and male gametocytes in mice infected with either WT or *PbBrca2*-KO1 parasites. Percentages of female (pink) and male (blue) gametocytes are shown. **D** Exflagellation ratios were measured for in vitro cultures using blood from five mice infected with WT or *PbBrca2*-KO1 parasites and with approximately 10% parasitemia. **E** Ookinete counts for in vitro cultures after 24 h of incubation at 19 °C in ookinete culture medium using blood from five mice infected with WT or *PbBrca2*-KO1 parasites and with approximately 10% parasitemia. Data are plotted as bar graphs. *F*-test followed by Student’s *t*-test was used to compare the mean values of gametocytemia, female and male ratio, exflagellation, ookinete number. Error bars indicate SD. * *P* < 0.05, ** *P* < 0.01,* N.S.* non-significant (vs. WT parasites).* RBC* Red blood cells; for other abbreviations, see Figs. 1 and 2

The *PbBrca2*-KO1 and *PbBrca2*-KO2 clones were similar in terms of the severity of parasitemia and oocyst number. Hence, we analyzed the *PbBrca2*-KO1 clone in the subsequent experiments. We enumerated gametocytemia and the proportions of female and male gametocytes using blood smears from *PbBrca2*-KO or WT parasite-infected mice. There were no apparent differences between the *PbBrca2*-KO and WT parasites in terms of gametogony morphology (Fig. 4a). However, the *PbBrca2*-KO parasites induced less gametocytemia than the WT parasites (Fig. 4b). The proportions of female and male gametocytes differed between the WT and *PbBrca2*-KO parasites (Fig. 4c). Statistical analysis also confirmed that the total number of female gametocytes was lower and the total number of male gametocytes higher in mice infected with *PbBrca2*-KO parasites compared to those infected with WT parasites (Table 2). However, the exflagellation ratios for WT and *PbBrca2*-KO parasites were comparable. Therefore, male gametocyte maturation was considered morphologically normal in the *PbBrca2*-KO parasites (Fig. 4d). Ookinete formation was also reduced in the *PbBrca2*-KO parasites, but there were no apparent morphological differences between the *PbBrca2*-KO and WT ookinetes (Fig. 4a, e).

**Table 2:** Total numbers of female and male gametocytes^a^

Parasite	Female	Male	χ^2^-test
WT	230 (46 ± 17.89)	57 (11.4 ± 4.51)	*P* < 0.01
*PbBrca2*-KO1	173 (34.6 ± 7.23)	95 (19 ± 2.35)	

The numbers in parentheses indicate the mean ± SD. For abbreviations, see Table 1

^a^The total numbers of female and male gametocytes were calculated for four infected mice (see Fig. 4c)

### *PbBrca2*-KO parasite oocysts presented with a developmental abnormality

**Fig. 5:**
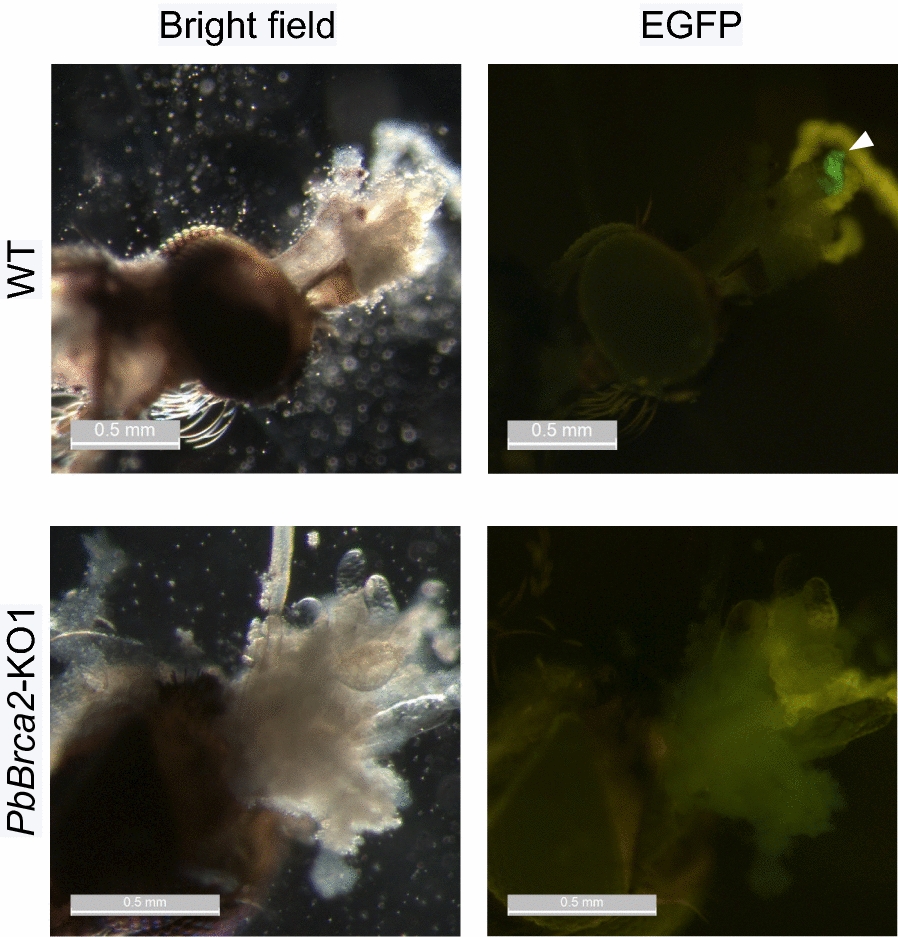
PbBrca2 is essential for sporozoite development in the salivary gland. *Anopheles stephensi* mosquitoes were fed on infected mice, and the sporozoites were microscopically examined at day 28 post-infection. Arrowheads indicate enhanced green fluorescent protein (*EGFP*)-positive sporozoites, which were detected for WT parasites only. For other abbreviations, see Figs. 1 and 2

**Fig. 6:**
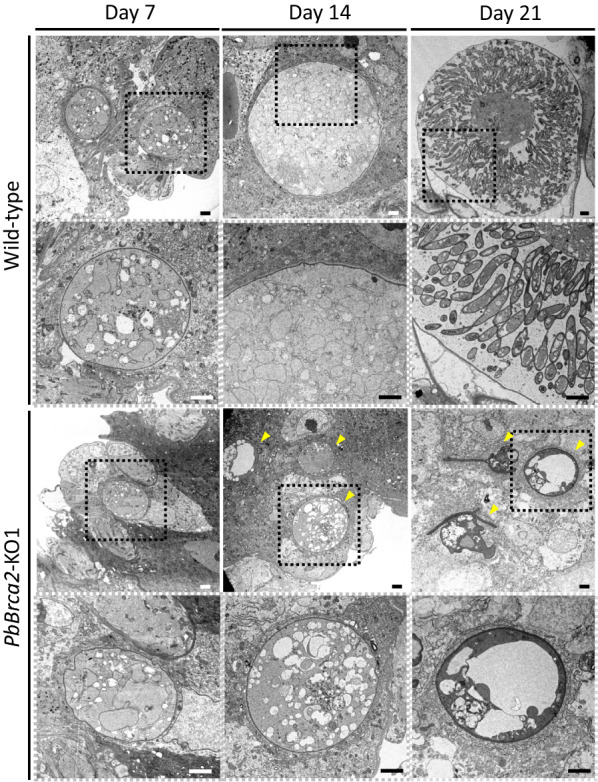
PbBrca2 is essential for sporogony. Ultrastructural analyses of WT and *PbBrca2*-KO1 oocytes at 7, 14, and 21 days post-infection. WT exhibited normal sporogony, whereas *PbBrca2*-KO1 oocysts were small and had indeterminate morphology (day 7). *PbBrca2*-KO1 parasites did not form sporozoites, and in some cases their oocysts shrank and became vacuolated (day 14). *PbBrca2*-KO1 oocysts showing notable shrinkage (day 21). Image parts delineated by white dotted lines (lower panels) are magnifications of image parts delineated by black dotted lines (upper panels). Arrowheads indicate abnormal parasite development. Scale bar = 1 μm. For abbreviations, see Figs. 1 and 2

Mosquitoes infected with *PbBrca2*-KO parasites could not transmit them to their secondary hosts. Furthermore, sporozoites were absent from the salivary glands of these mosquitoes at day 28 post-infection (Fig. 5). Thus, we examined the morphology of *PbBrca2*-KO parasite oocysts and sporozoites in the mosquito midguts (Fig. 6). An ultrastructural analysis disclosed that *PbBrca2*-KO formed oocysts in the midguts on day 7 post-infection. By contrast, *PbBrca2*-KO revealed no sporozoite formation in the oocysts on day 14 or 21 post-infection. *PbBrca2*-KO parasites showed undeveloped oocysts after day 7 post-infection. On day 14 post-infection, the oocysts shrank and vacuoles appeared in them. Certain mosquitoes shed oocysts from their midgut lumens on day 14 (data not shown). At day 21 post-infection, the *PbBrca2*-KO oocysts were vacuolated and the oocyst capsules shrank. These results indicate that *PbBrca2*-KO ookinetes can differentiate into oocysts but not sporozoites in mosquito midguts. Therefore, *PbBrca2*-KO cannot undergo sporogony and has a defective life cycle.

## Discussion

In the present study, we identified the recombinase-regulating protein PbBrca2 and determined the phenotype of *PbBrca2*-KO parasites. PbBrca2 is well conserved among various *Plasmodium* species. BRC repeats of human BRCA2 could not interact with *S. cerevisiae* Rad51 [18]. In the present study, BRC repeats of PbBrca2 interacted with PbRad51 but not with human or *S. cerevisiae* Rad51 (as confirmed by the mammalian two-hybrid assay). We also attempted to assess the interaction between PbBrca2 BRC repeats and PbRad51 via immunoprecipitation, but could not detect the interaction, probably because of the low expression of *Plasmodium* proteins in the human cell line. Furthermore, using in silico analysis, we assessed the importance of the Rad51 amino acids M158, I205, M251, and L255 because they interact with the phenylalanine residues F1524 and F1546 in human BRC repeat 4. Their substitution reduced the number of contacts between BRC repeat 4 and Rad51. *Plasmodium* Rad51 forms homomultimers and mediates strand exchange [4, 33]. Homology between *P. berghei* and human Rad51 is > 80% but the chemical properties of the four amino acids near the BRC repeat binding site differ between human and *P. berghei* Rad51. Moreover, the amino acid sequences of the BRC repeats also differ between human and *P. berghei* Brca2. For these reasons, the interactions between Rad51 and Brca2 have uniquely evolved in each species and differ between humans and *Plasmodium*. This discrepancy might facilitate the development and application of anti-*Plasmodium* drugs. We also tested the interaction activity between PbBrca2 BRC repeats and PbDmc1, as it was reported that human BRCA2 interacted with Dmc1 via BRC repeats [9]. However, we detected no such interaction activity in *P. berghei*. Existence of the DMC1-binding domain in human BRCA2 is controversial. Another study reported a DMC1-specific binding domain and the absence of any interaction with DMC1 via BRC repeats containing peptides [10]. Thus, interaction analysis using partial PbBrca2 peptides is required to identify the Dmc1-binding domain in *Plasmodium* Brca2.

Here, we endeavored to establish why relatively few *PbBrca2*-KO oocysts were found in the mosquitoes. The *PbBrca2*-KO parasites also presented reduced numbers of gametocytes. The female gametocyte ratio decreased whereas the exflagellation ratio (male gametocyte number) did not. *Brca2* was upregulated in female gametocytes but only weakly expressed in male gametocytes [32]. These results suggest that female gametocyte differentiation was altered or destabilized in *PbBrca2*-KO parasites. Knocking out *PbBrca2* produced morphologically normal male gametocytes, but their function could not be well assessed. To understand the function of Brca2 in male gametocytes, cross-fertilization assays using male-specific sterile lines, such as *CDPK4*-KO or *Map2*-KO parasites, are warranted [34, 35]. Moreover, for *PbBrca2*-KO parasites, the number of gametocytes was reduced and there were even fewer post-meiosis ookinetes. PbBrca2 may contribute to HR during meiosis. BRCA2 mutations in mice and zebra fish led to infertility by inhibiting gamete development and inducing gamete death [36, 37]. Hence, these might induce atypical meiosis and parasite death. A few *PbBrca2*-KO parasites were nonetheless able to form oocysts. Transmission electron microscopy revealed no sporogony. *PbBrca2*-KO parasite differentiation was terminated at ~ 7 days after WT parasite infection. At day 14 post-infection, vacuoles appeared in the oocysts and the latter began to degrade. Thus, PbBrca2 is also required for sporozoite development.

Some results of the present study align with those previously reported for the *PbDmc1*-KO parasite [5]. *Plasmodium* Dmc1 is a meiosis-specific recombinase [5]. In humans, DMC1 contributes to HR and is regulated by BRCA2 during meiosis [7]. The *PbDmc1*-KO parasites presented with relatively reduced oocyst and sporozoite numbers. Moreover, their phenotype was less pathological than that of our *PbBrca2*-KO parasites. However, the *PbDmc1*-KO parasites could nonetheless differentiate into sporozoites. In contrast, the *PbBrca2*-KO parasites displayed reduced numbers of female gametocytes, ookinetes, and oocysts, and presented with no sporozoite development. They also proliferated only very slowly at the blood cell stage. It was predicted that PbDmc1 only plays a role during meiosis, and that its KO would have a limited effect. PbBrca2 might have a broader function than Dmc1. Moreover, *PbBrca2* KO influenced all the *Plasmodium* life cycle stages. Further investigations on the function of PbBrca2 in meiosis are required to determine whether the roles of Brca2 are more important than those of Dmc1 in *Plasmodium*.

In humans, BRCA2 plays pivotal roles in HR repair when DNA is damaged by DSBs, and in meiotic HR via RAD51 binding [38]. We observed a slow progression of the blood stages of the *PbBrca2*-KO parasites, with an increased proportion of parasites at the ring stage and a decreased proportion at the trophozoite stage compared to those observed for the WT parasites. It is possible that parasitemia was lower in the case of *PbBrca2*-KO parasites than WT parasites due to the increased induction of life cycle arrest resulting from damage to DNA during stages of asexual reproduction. Responses to DNA damage were not examined in the present study. However, the contribution of PbBrca2 to HR after DNA DSBs could be examined in a future study by evaluating the sensitivity of PbBrca2 to agents inducing DNA damage during asexual reproduction.

## Conclusions

Here, we identified *Plasmodium* Brca2 harboring BRC repeats and OB and tower domains, which are sequence fingerprints of BRCA2. *Brca2* KO in *P. berghei* resulted in reduced parasitemia, with an increase in counts at the ring stage and a decrease at the trophozoite stage, a decrease in oocyst formation, a decrease in the number of oocytes and a reduction in ookinete development. Sporogony did not progress under *Brca2* KO. The results of the present, and previous, studies suggest that Brca2 contributes to HR during meiosis. The absence of Brca2 had an effect on the sporogony and nearly every other developmental stage of *Plasmodium*. Brca2 might be a potential anti-*Plasmodium* drug target. To the best of our knowledge, this study is the first step in elucidating the function of Brca2 in *Plasmodium*, and the results presented here help clarify the mechanism of meiosis in this pathogen.

## Supplementary Information

**Additional file 1: Figure S1. **Alignment of Brca2 amino acid sequence among *Plasmodium* spp. Alignment of *Plasmodium berghei, Plasmodium yoelii, Plasmodium falciparum, Plasmodium vivax, Plasmodium ovale, Plasmodium malariae,* and *Plasmodium knowlesi* Brca2. Red and blue boxes indicate BRC repeats and OB and tower domains in *P. berghei* Brca2, respectively.

**Additional file 2: Figure S2. **Three-dimensional structure of DNA-binding domains in *Plasmodium berghei* Brca2.** A** Three-dimensional structures of OB, tower, and DNA-binding domains in *P. berghei* Brca2 were predicted with AlphaFold2 and compared against human BRCA2 domains (PDB ID 1MIU).** B** Three-dimensional structures of OB and tower domains in *P. berghei* Brca2 were compared against the typical OB domain of human replication protein A1 (PDB ID 4O0A).

**Additional file 3: Figure S3. **A human and *P. berghei* Rad51 alignment. Red characters indicate amino acid residues interacting with vital phenylalanine residues F1524 and F1546 in BRC repeat 4 in human Rad51. Blue characters in *P. berghei* Rad51 indicate the amino acid residues that differ from the human Rad51 amino acid residues required for interaction with F1524 and F154 in BRC repeat 4.

## Data Availability

The datasets used and/or analyzed in the current study are available from the corresponding author upon reasonable request.

## Abbreviations

BRCA2: Breast cancer susceptibility protein 2
DMC1: DNA meiotic recombinase 1
DSB: Double-strand break
HR: Homologous recombination
KO: Knockout
OB: Oligonucleotide/oligosaccharide-binding
OKM: Ookinete culture medium
PCR: Polymerase chain reaction
Rad51: DNA repair protein Rad51 homolog 1
WT: Wild type

## References

[1] Ross LS, Fidock DA (2019). Elucidating mechanisms of drug-resistant *Plasmodium falciparum*. Cell Host Microbe.

[2] Skwarczynski M, Chandrudu S, Rigau-Planella B, Islam MdT, Cheong YS, Liu G (2020). Progress in the development of subunit vaccines against malaria. Vaccines.

[3] Lee AH, Symington LS, Fidock DA (2014). DNA repair mechanisms and their biological roles in the malaria parasite *Plasmodium falciparum*. Microbiol Mol Biol Rev.

[4] Roy N, Bhattacharyya S, Chakrabarty S, Laskar S, Babu SM, Bhattacharyya MK (2014). Dominant negative mutant of* Plasmodium* Rad51 causes reduced parasite burden in host by abrogating DNA double-strand break repair. Mol Microbiol.

[5] Mlambo G, Coppens I, Kumar N (2012). Aberrant sporogonic development of Dmc1 (a meiotic recombinase)-deficient *Plasmodium berghei* parasites. PLoS ONE.

[6] Kelso AA, Waldvogel SM, Luthman AJ, Sehorn MG (2017). Homologous recombination in protozoan parasites and recombinase inhibitors. Front Microbiol.

[7] Li Q, Engebrecht J (2021). BRCA1 and BRCA2 tumor suppressor function in meiosis. Front Cell Dev Biol.

[8] Andreassen PR, Seo J, Wiek C, Hanenberg H (2021). Understanding BRCA2 function as a tumor suppressor based on domain-specific activities in DNA damage responses. Genes.

[9] Martinez JS, von Nicolai C, Kim T, Ehlén Å, Mazin AV, Kowalczykowski SC (2016). BRCA2 regulates DMC1-mediated recombination through the BRC repeats. Proc Natl Acad Sci.

[10] Thorslund T, Esashi F, West SC (2007). Interactions between human BRCA2 protein and the meiosis-specific recombinase DMC1. EMBO J.

[11] Carreira A, Kowalczykowski SC (2011). Two classes of BRC repeats in BRCA2 promote RAD51 nucleoprotein filament function by distinct mechanisms. Proc Natl Acad Sci.

[12] Kirkman LA, Lawrence EA, Deitsch KW (2014). Malaria parasites utilize both homologous recombination and alternative end joining pathways to maintain genome integrity. Nucleic Acids Res.

[13] Kroeger PT, Drummond BE, Miceli R, McKernan M, Gerlach GF, Marra AN (2017). The zebrafish kidney mutant zeppelin reveals that brca2/fancd1 is essential for pronephros development. Dev Biol.

[14] Martin JS, Winkelmann N, Petalcorin MIR, McIlwraith MJ, Boulton SJ (2005). RAD-51-dependent and -independent roles of a *Caenorhabditis elegans* BRCA2-related protein during DNA double-strand break repair. Mol Cell Biol.

[15] Kojic M, Yang H, Kostrub CF, Pavletich NP, Holloman WK (2003). The BRCA2-interacting protein DSS1 is vital for DNA repair, recombination, and genome stability in *Ustilago maydis*. Mol Cell.

[16] Hartley CL, McCulloch R (2008). *Trypanosoma brucei* BRCA2 acts in antigenic variation and has undergone a recent expansion in BRC repeat number that is important during homologous recombination. Mol Microbiol.

[17] Genois M-M, Mukherjee A, Ubeda J-M, Buisson R, Paquet E, Roy G (2012). Interactions between BRCA2 and RAD51 for promoting homologous recombination in *Leishmania infantum*. Nucleic Acids Res.

[18] Jensen RB, Carreira A, Kowalczykowski SC (2010). Purified human BRCA2 stimulates RAD51-mediated recombination. Nature.

[19] Zimmermann L, Stephens A, Nam S-Z, Rau D, Kübler J, Lozajic M (2018). A completely reimplemented MPI bioinformatics toolkit with a new HHpred server at its core. J Mol Biol.

[20] Gabler F, Nam S, Till S, Mirdita M, Steinegger M, Söding J (2020). Protein sequence analysis using the MPI bioinformatics toolkit. Curr Protoc Bioinform.

[21] Nakai K, Horton P, Nakai K, Horton P (1999). PSORT: A program for detecting sorting signals in proteins and predicting their subcellular localization. Trends Biochem Sci.

[22] Nakai K, Kanehisa M (1992). A knowledge base for predicting protein localization sites in eukaryotic cells. Genomics.

[23] Jumper J, Evans R, Pritzel A, Green T, Figurnov M, Ronneberger O (2021). Highly accurate protein structure prediction with AlphaFold. Nature.

[24] Ishino T, Orito Y, Chinzei Y, Yuda M (2006). A calcium-dependent protein kinase regulates *Plasmodium* ookinete access to the midgut epithelial cell. Mol Microbiol.

[25] Yoshikawa Y, Morimatsu M, Ochiai K, Ishiguro-Oonuma T, Morioka R, Okuda K (2021). Identification of the core motif of the BRCA2 C-terminal RAD51-binding domain by comparing canine and human BRCA2. J Vet Med Sci.

[26] Soga A, Bando H, Ko-ketsu M, Masuda-Suganuma H, Kawazu S, Fukumoto S (2017). High efficacy in vitro selection procedure for generating transgenic parasites of *Plasmodium berghei* using an antibiotic toxic to rodent hosts. Sci Rep.

[27] Soga A, Shirozu T, Fukumoto S (2021). Glyoxalase pathway is required for normal liver-stage proliferation of *Plasmodium berghei*. Biochem Bioph Res Commun.

[28] Nakayama K, Kimura Y, Kitahara Y, Soga A, Haraguchi A, Hakozaki J (2021). Role of *Plasmodium berghei* ookinete surface and oocyst capsule protein, a novel oocyst capsule-associated protein, in ookinete motility. Parasit Vectors.

[29] Sasaki H, Sekiguchi H, Sugiyama M, Ikadai H (2017). *Plasmodium berghei* Cap93, a novel oocyst capsule-associated protein, plays a role in sporozoite development. Parasit Vectors.

[30] Sugiyama M, Machida N, Yasunaga A, Terai N, Fukasawa H, Ono HK (2021). Vaginal mucus in mice: developmental and gene expression features of epithelial mucous cells during pregnancy. Biol Reprod.

[31] Rajendra E, Venkitaraman AR (2010). Two modules in the BRC repeats of BRCA2 mediate structural and functional interactions with the RAD51 recombinase. Nucleic Acids Res.

[32] Howick VM, Russell AJC, Andrews T, Heaton H, Reid AJ, Natarajan K (2019). The malaria cell atlas: single parasite transcriptomes across the complete *Plasmodium* life cycle. Science.

[33] Bhattacharyya MK, nee Bhattacharyya Deb SB, Jayabalasingham B, Kumar N (2005). Characterization of kinetics of DNA strand-exchange and ATP hydrolysis activities of recombinant PfRad51, a *Plasmodium falciparum* recombinase. Mol Biochem Parasitol.

[34] Billker O, Dechamps S, Tewari R, Wenig G, Franke-Fayard B, Brinkmann V (2004). Calcium and a calcium-dependent protein kinase regulate gamete formation and mosquito transmission in a malaria parasite. Cell.

[35] Tewari R, Dorin D, Moon R, Doerig C, Billker O (2005). An atypical mitogen-activated protein kinase controls cytokinesis and flagellar motility during male gamete formation in a malaria parasite. Mol Microbiol.

[36] Rodríguez-Marí A, Wilson C, Titus TA, Cañestro C, BreMiller RA, Yan Y-L (2011). Roles of *brca2* (*fancd1*) in oocyte nuclear architecture, gametogenesis, gonad tumors, and genome stability in zebrafish. PLoS Genet.

[37] Sharan SK, Pyle A, Coppola V, Babus J, Swaminathan S, Benedict J (2004). BRCA2 deficiency in mice leads to meiotic impairment and infertility. Development.

[38] Sun Y, McCorvie TJ, Yates LA, Zhang X (2020). Structural basis of homologous recombination. Cell Mol Life Sci.

