# Supplementary material for: Plasmodium berghei Brca2 is required for normal development and differentiation in mice and mosquitoes
Source: Parasit Vectors. 2022 Jul 8;15:244. doi: 10.1186/s13071-022-05357-w (PMC9270840; doi:10.1186/s13071-022-05357-w)
Supplement: Supplementary file 1 — Additional file 1: Figure S1. Alignment of Brca2 amino acid sequence among Plasmodium spp. Alignment of Plasmodium berghei, Plasmodium yoelii, Plasmodium falciparum, Plasmodium vivax, Plasmodium ovale, Plasmodium malariae, and Plasmodium knowlesi Brca2. Red and blue boxes indicate BRC repeats and OB and tower domains in P. berghei Brca2, respectively. [file 13071_2022_5357_MOESM1_ESM.pdf]

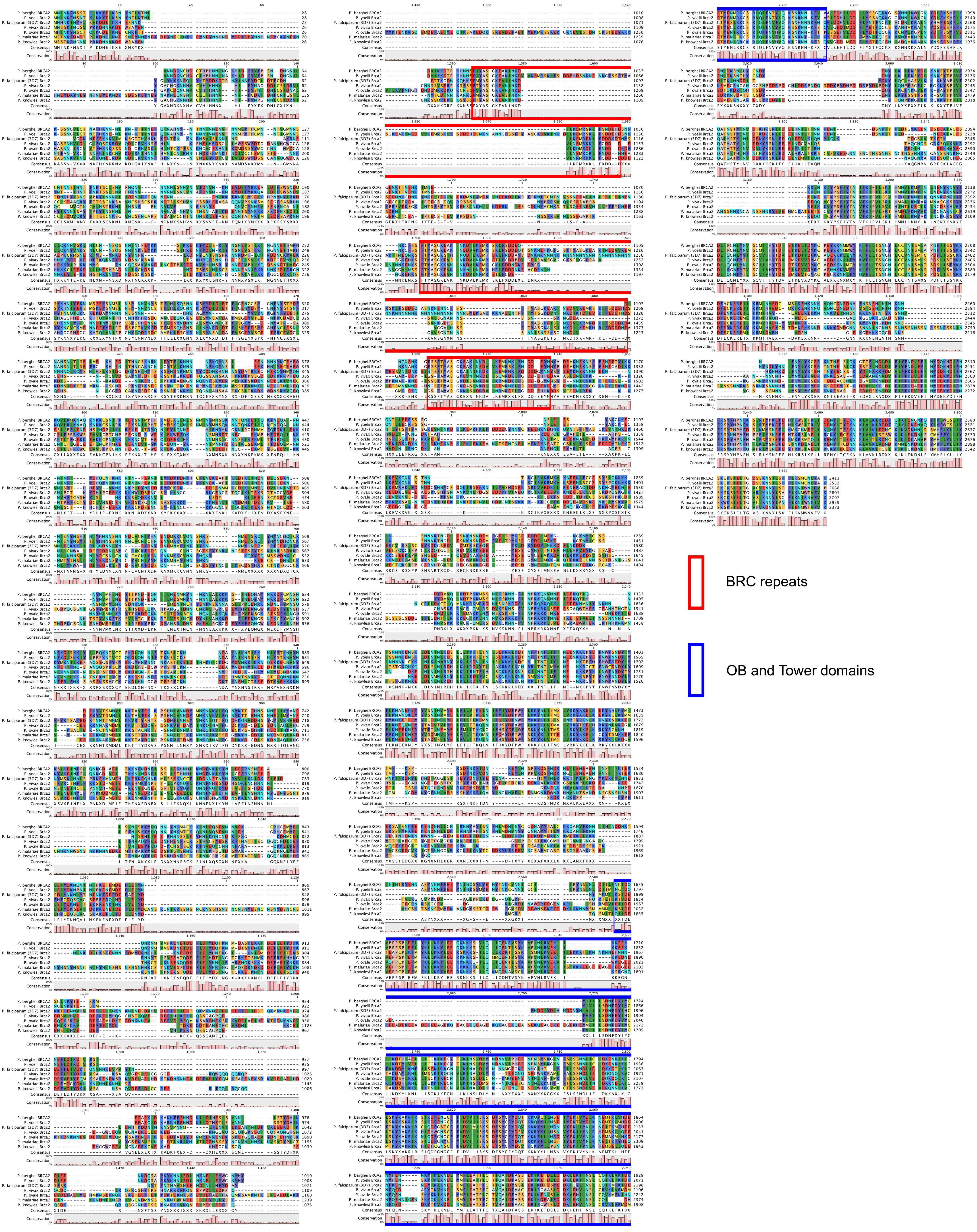

Additional file 1: Fig. S1 Alignment of Brca2 amino acid sequence among *Plasmodium* spp.

Alignment of *P. berghei, P. yoelii, P. falciparum, P. vivax, P. ovale, P. malariae,* and *P. knowlesi* Brca2.

Red and blue boxes indicate BRC repeats and OB and tower domains in *P. berghei* Brca2, respectively.
