# Supplementary material for: Plasmodium berghei Brca2 is required for normal development and differentiation in mice and mosquitoes
Source: Parasit Vectors. 2022 Jul 8;15:244. doi: 10.1186/s13071-022-05357-w (PMC9270840; doi:10.1186/s13071-022-05357-w)
Supplement: Supplementary file 2 — Additional file 2: Figure S2. Three-dimensional structure of DNA-binding domains in Plasmodium berghei Brca2. A Three-dimensional structures of OB, tower, and DNA-binding domains in P. berghei Brca2 were predicted with AlphaFold2 and compared against human BRCA2 domains (PDB ID 1MIU). B Three-dimensional structures of OB and tower domains in P. berghei Brca2 were compared against the typical OB domain of human replication protein A1 (PDB ID 4O0A). [file 13071_2022_5357_MOESM2_ESM.pdf]

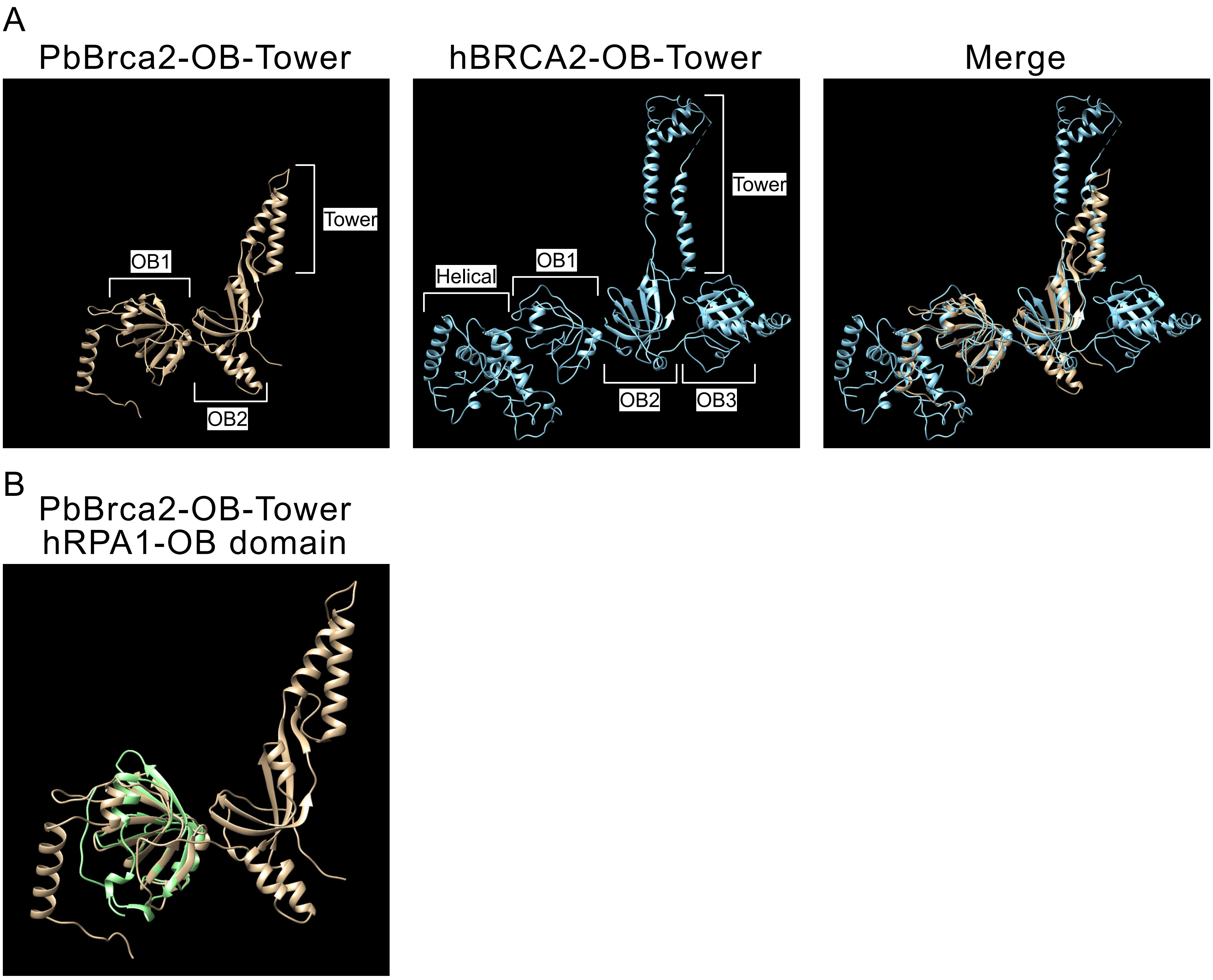

Additional file 2: Fig. S2 3D structure of DNA-binding domains in *Plasmodium berghei* Brca2. (**A**) 3D structures of OB, tower, and DNA-binding domains in *P. berghei* Brca2 were predicted with AlphaFold2 and compared against human BRCA2 domains (PDB ID: 1MIU). (**B**) 3D structures of OB and tower domains in *P. berghei* Brca2 were compared against typical OB domain of human replication protein A1 (PDB ID: 4O0A)
