# Supplementary material for: Plasmodium berghei Brca2 is required for normal development and differentiation in mice and mosquitoes
Source: Parasit Vectors. 2022 Jul 8;15:244. doi: 10.1186/s13071-022-05357-w (PMC9270840; doi:10.1186/s13071-022-05357-w)
Supplement: Supplementary file 3 — Additional file 3: Figure S3. A human and P. berghei Rad51 alignment. Red characters indicate amino acid residues interacting with vital phenylalanine residues F1524 and F1546 in BRC repeat 4 in human Rad51. Blue characters in P. berghei Rad51 indicate the amino acid residues that differ from the human Rad51 amino acid residues required for interaction with F1524 and F154 in BRC repeat 4. [file 13071_2022_5357_MOESM3_ESM.pdf]

```
hRad51          ----MAMQMQLEANADTSVEEES-----FGPQPISRLEQCGINANDVKKLEEAGFHTVEA 51
PbRad51         MKSANAKEDTISQTCDNSTTEEVDEHLYAGPLKIEQLLAKGFVKRDLELLKEGGLQTVEC 60
                     * :  :. ..*.*. **       **  *.:*   *:  .*:: *:*.*::***.

hRad51          VAYAPKKELINIKGISEAKADKILAEAAKLVPMGFTTATEFHQRRSEIIQITTGSKELDK 111
PbRad51         VAYAPMRTLCSIKGISEQKAEKLKKACKELCNSGFCNAIDYHDARQNLIKFTTGSKQLDA 120
                ***** : * .****** **:*:   . :*   ** .* ::*: *.::*::*****:**

hRad51          LLQGGIETGSITEMFGEFRTGKTQICHTLAVTCQLPIDRGGGEGKAMYIDTEGTFRPERL 171
PbRad51         LLKGGIETGGITELFGEFRTGKSQLCHTLAITCQLPIEQSGGEGKCLWIDTEGTFRPERI 180
                **:******.***:********:*:*****:******::.*****.::***********:

hRad51          LAVAERYGLSGSDVLDNVAYARAFNTDHQTQLLYQASAMMVESRYALLIVDSATALYRTD 231
PbRad51         VAIAKRYGLHPTDCLNNIAYAKAYNCDHQTELLIDASAMMADTRFALLIVDSATALYRSE 240
                :*:*:****  :* *:*:***:*:* ****:** :*****.::*:*************::

hRad51          YSGRGELSARQMHLARFLRMLLRLADEFGVAVVITNQVVAQVDGAAMFAADPKKPIGGNI 291
PbRad51         YTGRGELANRQSHLCRFLRGLQRIADIYGVAVIITNQVVAKVDAMSMFGGHEKIPIGGNI 300
                *:*****: ** **.**** * *:** :****:*******:**. :**... * ******
```

Additional file 3: Fig. S3 Human and *P. berghei* Rad51 alignment.

Red characters in human Rad51 indicate amino acid residues interacting with vital phenylalanine residues F1524 and F1546 in BRC repeat 4. Blue characters indicate the amino acid residues that differ from the human Rad51 amino acid residues required for interaction with F1524 and F154 in BRC repeat 4.
